# Supplementary material for: Diagnostic accuracy of a smartphone-based device (VistaView) for detection of diabetic retinopathy: A prospective study
Source: PLOS Digit Health. 2024 Nov 8;3(11):e0000649. doi: 10.1371/journal.pdig.0000649 (PMC11548746; doi:10.1371/journal.pdig.0000649)
Supplement: S1 File — (PDF) [file pdig.0000649.s001.pdf]

DIAGNOSTIC ACCURACY OF A SMARTPHONE BASED DEVICE (VISTAVIEW) FOR DETECTION OF DIABETIC RETINOPATHY:
A Prospective Study

Diabetic retinopathy (DR) is a leading cause of blindness **globally**. The gold standard for DR screening is stereoscopic fundus photography. VistaView is a smartphone-based retinal camera. The aim of this study was to evaluate the diagnostic accuracy of VistaView in detecting DR.

1428 fundus images from 371 patients with both cameras.

A prospective Study

December 2021 and June 2022

Karachi, Pakistan

Images graded by two graders based on the International Classification of Diabetic Retinopathy (ICDR)

GOLD STANDARD

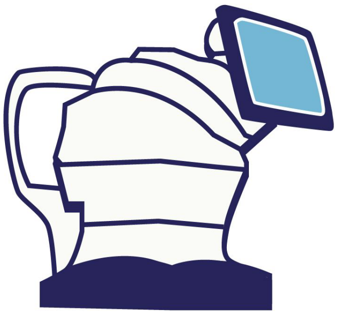

VISTAVIEW

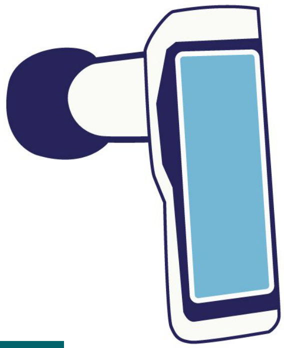

DIAGNOSTIC ACCURACY

	DR	RDR
✓ Sensitivity	69.9%	69.7%
✓ Specificity	92.9%	94.2%

Gold Standard

85% ICC of DR grades

79% Maculopathy grades

0.68 Cohen's Kappa for DR grades

0.65 Maculopathy Grades

VistaView

78% ICC of DR grades

66% Maculopathy grades

0.61 Cohen's Kappa for DR grades

0.49 Maculopathy Grades

CONCLUSION:

VistaView offers high diagnostic accuracy for DR screening with comparable agreement levels between graders using fundus camera. Therefore, VistaView may be used as a screening tool for DR.
