# Supplementary material for: Diagnostic accuracy of a smartphone-based device (VistaView) for detection of diabetic retinopathy: A prospective study
Source: PLOS Digit Health. 2024 Nov 8;3(11):e0000649. doi: 10.1371/journal.pdig.0000649 (PMC11548746; doi:10.1371/journal.pdig.0000649)
Supplement: Attachment — Submitted filename: Response to Reviewers.docx [file pdig.0000649.s003.docx]

Dear Editor,

Thank you for your consideration for our manuscript “Diagnostic Accuracy of a Smartphone-Based Device (VistaView) for Detection of Diabetic Retinopathy: A Prospective Study”.

We have carefully addressed each comment and made the required amendments. Below is a list of each comment along with its corresponding response.

Sincerely,

Dr. Rehman Siddiqui

| **Additional Editor Comments** | **Response to Reviewers** | **Line number in track changes file** |
| --- | --- | --- |
| I suggest a review of English and text flow. | Reviewed and revised! |  |
| Line 162: Is 59 the mean age of the males specifically or the overall cohort? | 59 is the mean age of the entire cohort. The statement has been amended. | 198 |
| Comorbidities: Are there any reported comorbidities among the participants? | No, we did not report any comorbidities. |  |
| Capturing Protocol: What was the image-capturing protocol? Was it 1 image or 2 images per eye?  * How was the issue of a single bad-quality image in a set handled?  *How was the labeling performed—at the image level, eye level, or patient level? | Images were taken by trained technicians and reviewed on screen immediately. If image was considered poor quality, image acquisition was repeated upto maximum of 3 times. Best acquired image for kept for grading.  The labeling was performed at the image level. |  |
| Referable Criteria: Please clarify the referable criteria. Is it ICDR >1 and/or macular edema? | Yes, referable criteria is the stage moderate NPDR or higher, and/or maculopathy. That is indeed ICDR >1. This has been added to the manuscript. The definition of maculopathy has also been added. | 190  160-161 |
| Handling Low-Quality Images: How were low-quality images managed between devices for statistical analysis?  * It would be beneficial to describe the quality parameters across demographics, diabetic retinopathy (DR), and maculopathy severity between devices | Paired images were removed if either device image was ungradable.  Quality parameters were recorded as descriptive statistics as a secondary outcome for each grader – a ground truth was not established for quality parameters as they were very subjective – therefore there are no final quality grades for DR and maculopathy severity levels. |  |
| I suggest reporting the results for the ICDR scores grading between the devices, as the labeling grading was conducted instead of just any DR and Referable DR. | Thank you for your suggestion. We have reported percentages for the ICDR scores (no retinopathy; S0, mild NPDR; S1, severe NPDR; S3, PDR; S4) observed with both devices. To make it clinically applicable, we have performed the statistical analysis for diagnostic accuracy for any stage of DR and referable DR. |  |
| Image Quality Reporting: Why were image quality reports done by individual graders instead of by consensus and adjudication, as with DR scores? | Image Quality Reporting was highly subjective and it was decided to only report descriptive statistics for this parameter; therefore consensus and adjudication was not performed. |  |
| False Positives and False Negatives: Please report the false positive and false negative rates for each classification. | Values for fasle positive and false negative rates have been added to Table 3. | 251 |
| Additionally, I recommend including a statistical analysis to compare the performance of the devices, demographic, and associated factors. | Statistical analysis of devices’ performance has been conducted. Demographics have been described. |  |
| How do you justify the low low sensitivity of this device? I suggest expanding the discussion. | 1. Image quality 2. Media opacities | Added to discussion 311-312  319  322-330 |
|  |  |  |
| **Reviewer #1** |  |  |
| 1) Although the sensitivities found by the authors are lower than the recommended 80%, the authors conclude that "The vistaview offers high diagnostic accuracy for DR screening and can be used as a screening tool in LMIC". This conclusion is not based on the data. Please reconsider. | This comment stands correct and we have amended the statement in the conlucison. | 65 |
| 2) The sensitivities reported here are lower than for other handheld cameras in the literature. Please discuss. | Statement added, which leads into the discussion about the limiting factors of the VistaView which may affect its sensitivity.  Additionally, some studies reporting diagnostic accuracy of other handheld cameras excluded patients with media opacities which may reflect in higher diagnostic accuracies. We included such patients to represent real-world settings. Furthermore, since patients in our study were screened at a tertiary eyecare center, there were relatively higher number of media opacities. | 311-312  322-330 |
| 3) The discussion is a bit lengthy and could be shortened. | Thank you for the suggestion. We have tried to make the discussion as succinct as possible while covering all important points. |  |
| 4) Suggest to present the results on image gradeability before the accuracy data. | Thank you for your suggestion. As image gradeability was not our primary endpoint, we have presented it after the accuracy data. |  |
| 5) The manuscript would benefit from native speaker English review. | Noted and reviewed, thank you! |  |
|  |  |  |
| **Reviewer #2** |  |  |
| 1) Add a Detailed Description of the Research Object: It is recommended to add a detailed description of the research object in the research methods section, including key information such as inclusion and exclusion criteria, so that readers can have a more comprehensive understanding of the characteristics and representativeness of the research object. | Thank you for your comment! Research objectives are already mentioned right above methods section.  Inclusion and exclusion criteria are mentioned in the methods section. | 123-125  129-133 |
| 2) Discuss the Limitations and Future Directions of the Research: In the discussion section, it is recommended that the author discuss the limitations of this study and possible future research directions. For example, the applicability of VistaView in different populations, comparative studies with other screening tools, and DR screening methods based on artificial intelligence technology can be explored. This helps readers to have a more comprehensive understanding of the value and significance of this study and provides useful references for future research. | Thank you for your comment. The limitations of the study are already discussed in the discussion section.  Future research directions have also been discussed – with regards to populations in LMIC, and use of AI for DR screening. Comparitive studies with similar screening tools have been discussed. | 393  385-391 |
| All Subjects Underwent Mydriasis for Standard Color Fundus Photography: What is the significance of mydriasis? What is the value of promoting this method? | The VistaView is a mydriatic camera – therefore acquisiton of good images requires mydriasis. This makes dilation an important step when using this camera. |  |
| Clarification of "Maculopathy": Does “Maculopathy” refer to diabetic macular edema (DME) or does it include all macular lesions? | The definition of maculopathy has been added. | 160 |
| Terminology: The term “colour fundus photography” should not be abbreviated to “fundus photography.” | Thank you for your comment. This correction has been made throughout the manuscript. |  |
